# Too Many Males or Too Many Females? Classroom Sex Ratio, Life History Strategies and Risk-Taking Behaviors

**DOI:** 10.1007/s10964-022-01635-z

**Published:** 2022-06-01

**Authors:** Javier Salas-Rodríguez, Luis Gómez-Jacinto, Isabel Hombrados-Mendieta, Natalia del Pino-Brunet

**Affiliations:** Department of Social Psychology, Social Work and Social Services, and Social Anthropology, University of Málaga, Málaga, Spain

**Keywords:** Evolutionary psychology, Classroom sex ratio, Life history strategy, Risk-taking behaviors, Multilevel modeling, Adolescents

## Abstract

Prior research finds that sex ratio, defined as the proportion of males and females in a given context, is related to engagement in risk-taking behaviors. However, most research operationalizes sex ratio at a local context (e.g., regional or county), which fails to reflect with precision the sex ratios contexts of individuals at a closer level. Furthermore, the relationship between sex ratio and risk-taking behaviors may be affected by individuals’ life history strategy, with previous studies showing fast life history strategies linked to risk-taking behaviors, compared to slow life history strategies. The present study analyzes the relationship between classroom sex ratio and risk-taking behaviors and the interaction between classroom sex ratio and life history strategy in adolescents. The sample comprised 1214 participants nested in 57 classrooms, 49.75% females, 91.5% Spanish and a mean age of 16.15 years (*SD* = 1.23, range 14–21). Results from multilevel modeling showed a negative relation between classroom sex ratio and risk-taking behaviors in female adolescents with faster life history strategy. By contrast, classroom sex ratio in male adolescents related positively to risk-taking behaviors but did not interact with life history strategy. These findings underscore the importance of studying proximate sex ratio on risk-taking behaviors in adolescents and underline its potential influence in the development and expression of life history strategies.

## Introduction

Risk-taking behaviors are currently among the main public health concerns in adolescents. Unprotected sex, drug abuse, dangerous driving, self-harming behaviors and/or aggressive behaviors are examples of risk-taking behaviors with potential negative effects on adolescents’ health and adjustment (Willoughby et al., 2021). These behaviors usually appear during the developmental stages of middle and late adolescence (Mata et al., 2016), and they are related to significant figures of external deaths in this population group (Ward et al., 2021). The potential negative effects of risk-taking behaviors have led research to take on a psychopathological approach on their study and conceptualize them as maladaptive outcomes to stressful environments (Nigg, 2017). However, from the evolutionary framework of risky adolescent behavior, engaging in such type of conducts is not necessarily understood as maladaptation. More specifically, risk-taking behaviors can exert potential functional and adaptive outcomes for adolescents’ survival and reproduction (e.g., accessing resources, status, or securing a partner), despite potential negative costs (Ellis et al., 2012). The evolutionary framework of risky behaviors links these behaviors to the development of individual life history strategies, related to patterns of resource allocation to cover basic needs of reproduction, parenting and growth of the individual (Wang et al., 2016), and to sex ratio, which is defined as the proportion of males and females in a given context (Emlen and Oring, 1977). However, few studies have analyzed the joint role of life history strategies and sex ratio and their relationship with risk-taking behaviors in adolescents. The present study aims to narrow this gap and therefore analyzes the interaction between life history strategies and classroom sex ratio on risk-taking behaviors in adolescents.

### Sexual Selection Theory

Sexual selection, along with natural selection, is one of the driving forces for the evolution of species. Sexual selection occurs through competition between same-sex individuals, generally males, in order to access the opposite sex (intrasexual competition), and selection by one of the sexes, generally females, of individuals of opposite sex (intrasexual selection) (Darwin, 1871). To explain these sex differences in reproductive strategies, the parental investment theory is suggested (Trivers, 1972). This theory defines parental investment as “any investment by the individual in its offspring that increases the offspring’s chances of surviving (and hence reproductive success) at the cost of the parent’s ability to invest in other offspring” (p.136). More specifically, human females are the sex that provides the most minimum physiological demands for reproduction and offspring survival (e.g., gestation, lactation or infant care in mammals). Furthermore, females have lower potential reproductive capacity compared to males and represent a limited resource (Trivers, 1972). Offspring survival also plays a key role for ultimate reproductive success in females, which promotes higher avoidance of risk-taking behaviors in females that could imply potential injury or death (Campbell, 2013). Alternatively, males’ potential reproductive capacity depends on the number of females they can access, thus leading them to compete for female partners in an effort to secure their individual reproductive success (Geary et al., 2004). This male-male competition occurs mainly through agonistic behaviors (fighting, chasing or displaying) aimed at deterring rivals, and courtship behaviors to attract potential mates (Weir et al., 2011). This provides successful males with higher potentiality to have larger numbers of offspring. In fact, since ancient times, males have fought to gain access to mates thus developing traits that contribute to their success in contest competition (Hill et al., 2013).

An evolutionary approach on risk-taking behaviors can help explain the adaptive value of these behaviors, as well as their higher expression in males. Additionally, there is an evolutionary explanation known as the “good genes hypothesis” (Zahavi, 1975), which proposes that females choose mates based on males’ genetic quality. Selecting mates with good genes will provide the offspring with relevant evolutionary mechanisms such as adaptability, good health or genetic variability (Gangestad et al., 2007). This is why risk-taking behaviors, despite the potential negative costs for individuals who exhibits them, can also contribute to the bearers’ fitness. In this sense, risk-taking behaviors can act as an honest indicator of good genes in males and which is highly valued by females (Kelly and Dunbar, 2001). Furthermore, females also choose their partners based on their ability to be good providers (Marlowe, 2003). In fact, the selection of mates considered good providers promotes higher intrasexual competition in males, with some studies suggesting that this contest competition could have been the main sexual selection mechanisms in males (Puts, 2010). Successful males in such contest competition will gain social status and access to resources, which will in turn contribute to their higher reproductive success (Hopcroft, 2006). It is therefore in this context of contest competition that risk-taking behaviors become a means through which males can exhibit formidability and gain social status against their potential rivals (Fessler et al., 2014). Finally, the choice of good fathers for helping in raising the offspring is another valuable factor considered by females, and which contrasts traits attributed to good provisioning skills, mainly based on intrasexual competition (Lu et al., 2015). This means that both contest competition and risk-taking behaviors would be traits opposed to good father attributes. In fact, good father attributes tend to show in species where males have higher reproductive success when involved in the care of their offspring than when they compete with other males (Lu et al., 2015). However, it is possible to see both reproductive strategies combined in one species, such as in humans (Geary et al., 2004).

Despite mate choice being mainly females’ decision, males also show preferences in their mates’ choices. More specifically, fertility and good genes in females are highly valued characteristics by males and are assessed based on physical traits related to females’ attractiveness (Thornhill & Gangestad, 1999). Fidelity and being a good mother are also qualities that male wish for in their partners, both addressed to easing paternal uncertainty and ensuring maternal investment (Thornhill & Gangestad, 1999). Finally, the ability to provide resources is also a highly valued trait in females, particularly in modern economies where women are fully involved in paid jobs (Lu et al., 2015). As a result, females also compete socially between them, particularly when it comes to breeding sites, reproduction opportunities, belonging to breeding groups and social status within these groups (Clutton-Brock & Huchard, 2013). That is, both reproductive strategies of contest competition as well as mate choice are highly widespread in males and females, with differences in these strategies being quantitative rather than qualitative (Campbell, 2013).

### Life History Theory

One important aspect from evolutionary theory is the analysis of individuals’ survival and reproduction strategies, which relates to life history strategy, a mid-level evolutionary theory that analyzes how organisms allocate efforts to meet a variety of survival and reproduction demands throughout their life cycles (Del Giudice et al., 2016). According to this theory, individuals must perform trade-offs when allocating finite energy and resources towards growth, maintenance, reproduction, and parental care. Moreover, using these trade-offs varies based on the demands of the closest environment, mainly in the stages of growth and development during childhood (Belsky et al., 2012). Throughout evolutionary history, the relation between the conditions of environments and life history strategies settled and this relation still determines organisms’ responses to their current environment (Pepper & Nettle, 2017). These environmental constraints which determine life history strategies are environmental unpredictability, related to morbidity-mortality (e.g., diseases, wars and violence), and environmental harshness, derived from resource scarcity (e.g. famine and economic recession) (Ellis et al., 2009). Due to individual differences in the exposure to the environmental cues of unpredictability and harshness, individuals’ trade-off decisions will differ, and can broadly be divided into those who allocate bio-energetic resource towards reproduction or early reproduction (i.e., fast life history strategists) and those who invest mainly in development and maintenance, including parenting (i.e., slow life history strategists) (Ellis et al., 2009). In general, unpredictable and harshness environments promote the development of fast life history strategies, where present fitness is prioritized and can be seen in a variation of physiological and psychological characteristics such as faster development, earlier sexual initiation, shorter-term partners, higher number of offspring that receive less investment, shorter-term approaches, higher immediate pleasure seeking, and higher risk engagement (Ellis et al., 2012). By contrast, predictable and controllable environments promote the development of slow life history strategies, which are characterized by the opposite pattern.

In essence, fast life history strategists tend characteristically to invest more time and energy in mating (reproduction), compared to slow life history strategists, who tend to invest more in growth, development and parental investment (Del Giudice & Belsky, 2011). As a result of such strategies, those individuals whose investment in health and longevity is more adaptive than the investment in reproduction (slow life history strategy) will be risk-averse, since they will have high future expectations and value potential losses; conversely, those individuals for whom it is more adaptive to invest in reproduction due to their lower expectations on a long and healthy life (fast life history strategy) will more prone to take risks since they have less to lose (Sear, 2020). Engaging in risk-taking behaviors would therefore be a trait of fast life history strategists, which has been previously observed in risky sexual behaviors (Belsky et al., 2010), aggression (Figueredo et al., 2018), and gambling and criminality (Mishra et al., 2017). These same findings have been observed in adolescents, where both males and females with fast life history strategies tend to show higher levels of engagement in risk-taking behaviors (Lehmann et al., 2018), and aggression and criminal conduct (Simmons et al., 2019).

Despite the key role of environmental harshness and unpredictability, there are other internal and external factors that can interact and impact the development of life history strategies. Both harshness and unpredictability can interact with internal body states and reinforce the individual’s development of life history strategy in the same direction (Chang et al., 2019). For instance, individuals who had been exposed to harsh and unpredictable environments during childhood and who had experienced adverse internal body states adjusted their pattern towards fast life history strategies in adolescence (Chang et al., 2019). It has also been seen that environmental unpredictability and harshness can interact with attachment between mother and child in the development of life history strategies (Lu et al., 2022). Results showed that insecure attachment strengthened the negative effect of childhood adversities over the development of a slow life history strategy; while in environments of secure attachment, such negative effect was mitigated (Lu et al., 2022).

It has also been seen that the development and expression of individuals’ life history strategies are not stable and may change throughout the life cycle due to changes in biological and social capital (e.g., offspring, relationships) as well as due to volatile information from the individual’s closest environment (Kubinski et al., 2017). This would be in line with the concept of “phenotypic plasticity”, which refers to individuals’ ability to adjust their life history strategies based on external and internal signals (Sear, 2020). When individuals’ closest environment lacks resources (harshness condition), they adjust their response and tend to spend more, in the case of fast life history strategists, or towards saving in the case of slow life history strategists (Griskevicius et al., 2013). Likewise, exposure to signs of mortality in adults (unpredictability condition) led individuals to feel an urge to postpone reproduction in slow life history strategists, as well as to invest more in their own embodied, and in fact, the opposite effect was observed in fast life history strategists (Griskevicius et al., 2011). Even females with fast life history strategies, when faced with signs of harshness, felt an urge to eat more, without restricting calories intake, thus promoting weight gain for the purpose of successful reproduction, while the opposite effect was seen in females with slower life history strategies (Hill et al., 2013).

Although risk-taking behaviors are generally linked with the development of fast life history strategies, the expression of these behaviors can be different in males and females. Variation in risk-taking behaviors due to life history strategy was found in young males but not in young females (Salas-Rodríguez et al., 2021). More specifically, engagement in risk-taking behaviors related to faster life history strategies in young males, while no differences in risk-taking behaviors based on life history strategy were found in females. These findings might imply differences between males and females when it comes to elements of life history strategy but also when it comes to the perception and response to the environment and which can affect the expression of certain behaviors, in this case risk-taking behaviors (Copping et al., 2017). In fact, although males are more responsive to contextual cues of mating and competition compared to females (Ellis et al., 2012), the adjustment of life history strategies to external cues seems to be more sensitive in females than in males (Richardson et al., 2017). Consequently, some authors recommend estimating the effect of life history strategy separated by sex, since sex differentiated pathways allow to obtain more information for a joint estimation in both sexes (Copping & Richardson, 2020).

### Operational Sex Ratio

So far it has been seen that trade-off decisions between the basic needs of survival and reproduction, encompassed in life history strategies can depend in part on individuals’ immediate ecological conditions, and that males and females may respond in different ways to these proximate external cues. The number of individuals competing for breeding partners is one of those ecological conditions that can moderate the intensity of reproductive competition in males and females, and which results from operational sex ratio (Clutton-Brock & Huchard, 2013). Operational sex ratio is defined as the ratio of sexually active males to sexually active females in a population at a given time and place (Emlen & Oring 1977). The theory of operational sex ratio proposes that sexual selection forces will be more frequent and intense in the sex with lower parental investment, since more individuals of that sex are ready and will compete for reproduction; at the same time, opposite-sex individuals become temporarily unavailable, given their higher parental investment (Emlen & Oring, 1977). Since skewed sex ratio affects the availability of potential mates, a competition between the members of the most abundant sex will increase (de Jong et al., 2012). However, other authors suggest that in skewed sex ratio contexts, intrasexual competition can be higher amongst the minority sex, given their potential benefits of accessing higher numbers of potential partners (Schacht et al., 2014). Furthermore, since males compete more intensely than females for mating, a skewed sex ratio results in a more dramatic intrasexual competition in males (Weir et al., 2011). Therefore, in more male-skewed sex ratios, males will invest more in competitive behaviors such as risk-taking behaviors, given their higher difficulty to access mates (Sng and Ackerman, 2020). In any case, sex ratio can also determine the intensity of mate competition in females and therefore affect their engagement in risk taking-behaviors (Campbell, 2013).

Some authors have suggested that operational sex ratio does not always determine the mating system of species and that both phenomena can be independent results from sexual selection (Willson & Pianka, 1963). Some recent models suggest that the intensity of intrasexual competition between males does not always depend on operational sex ratio but depends mainly on the potential costs linked to investing in a specific trait aimed at increasing mating rate (Kokko et al., 2012). This means that intrasexual competition may depend to a greater extent on how much individuals invest in a trait that increases mating success, regardless of the costs that such trait can cause (Kokko et al., 2012). As a result, intrasexual competition would be more intense when potential gains—number of available opposite-sex individuals—increases faster than the costs caused by a specific trait—the possibility of dying in competition.

Despite some theoretical objections, the role of sex ratio at local level (i.e., states, counties, municipalities) has been analyzed for a wide range of risk-taking behaviors. For example, US communities with male-skewed sex ratios showed a relation with higher levels of binge drinking in males but not in females (Aung et al., 2019). Additionally, countries with male-skewed sex ratio showed higher tendency towards extreme risk-taking behaviors such as male suicide attacks (Gibson, 2011). In fact, male-skewed sex ratio could be linked to historic Vikings raids, where low-status single men were more prone to risky behaviors in order to increase their wealth and status (Raffield et al., 2017). As a matter of fact, male-on-male violence and non-violent offending has been found to be more prevailing in male-skewed sex ratio municipalities in Stockholm metropolitan area, in Sweden (Filser et al., 2021) and campus with female-skewed sex ratios were associated with higher sexual activity in females (Uecker & Regnerus, 2010). In adolescents, higher propensity towards unprotected sex in females was related to female-skewed sex ratios at city-level in Brazil (Ramos et al., 2017). These studies are in line with the suggestion of higher intrasexual competition between the members of the majority group and which would lead to higher engagement in risk-taking behaviors. However, other studies that analyze the effect of sex ratio at local level found that it was the minority sex the one who exhibited more risk-taking behaviors when sex ratios were skewed. For example, at US county-level, males showed higher risk of infection by sexually transmitted diseases in female-skewed populations (Pouget, 2017), as well as higher rates of violent behavior (Schacht et al., 2016). Moreover, females who lived in male-skewed sex ratio wards in Northern Ireland showed higher risk of death by accidents, suicide, or alcohol abuse (Uggla & Mace, 2015). Finally, higher sexual activity and presence of sexually transmitted disease in male-skewed sex ratio communities in China was also found in females (Trent & South, 2012). In the case of adolescents, females showed higher sexual activity in male-skewed sex ratio communities in the US (Billy et al., 1994).

### Methodological Problems on the Study of Local Sex Ratio

Most studies that analyze sex ratio base their analyses on local sex ratio, with data aggregated to higher levels (i.e., nations, regions, or cities) (Pollet et al., 2017). However, analyzing sex ratio aggregated to higher levels can hide significant variations at a more proximate level. In other words, local sex ratio would not reflect the proportion of males and females in closest environments in a precise manner (Uggla & Mace, 2017). As a result, studies based on aggregated data might have fallen into the phenomenon of the “ecological fallacy”, where deductions about individuals are made based on data aggregated to higher levels (Pearce, 2000). Additionally, relations found between variables can be completely different based on each analysis level, that is, the same relation at individual level can show opposite results at national level (Kuppens & Pollet, 2014). Data collected from individuals’ situational sex ratios (e.g., workplaces, classrooms, hangouts) relates to individuals’ interactions with each other at its closest level and hence provides more accurate information on partner supply and demand, compared to local sex ratios (Filser & Preetz, 2021). In essence, operationalizing sex ratio in closely circumscribed social context levels is a more precise measurement of this variable compared to sex ratios operationalized at larger levels.

Despite its advantages, the effect of situational sex ratio has been analyzed to a lesser extent. However, there are some interesting results, such as the following cases: in the US, adolescent females showed higher probability of participating in serious violence when they were part of male-skewed friendship networks, while male adolescents who were part of female-skewed friendship networks showed lower probability (Haynie et al., 2007). In Germany, both male and female adolescent students showed higher risk of school violence in male-skewed classrooms, mainly in relation to injuries attributable to other students (Filser et al., 2022); and in the Netherlands, a higher relation between status and relational aggression in adolescent male students was found in classrooms with male-skewed sex ratios (Zwaan et al., 2013). However, in Israel, it was found that female-skewed classrooms sex ratio promoted academic improvement in both females and males, which was mediated by lower classroom disruption and violence (Lavy & Schlosser, 2011). Finally, in an experimental context, being part of the majority sex promotes aggressive behaviors towards desirable same-sex individuals and higher intrasexual competition, both in males and females (Moss & Maner, 2016).

At all events, even the studies that analyze sex ratio at situational level show contradictory results, thus making it difficult to stablish a clear relation between sex ratio and risk-taking behaviors. The absence of a clear pattern in the relation between risk-taking behaviors and sex ratio might be due, in part, to the existence of individual differences in the adjustment of sex ratio (Schacht et al., 2017). It is therefore necessary to analyze which factors are involved in individual differences in the adjustment of competition strategies between individuals based on sex ratio (Schacht et al., 2017). Likewise, life history strategy, given its role in individual differences in reproductive and competitive adjustment, as well as its ability to adjust to contextual variables, might be a relevant individual variable when it comes to explaining the association between sex ratio and risk-taking behaviors.

## Current Study

**Fig. 1:**
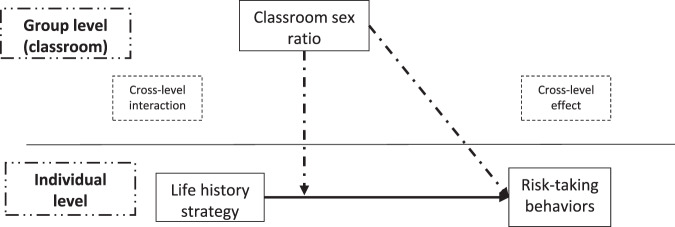
Multilevel Theoretical Model

Despite the relevance of sex ratio and life history strategies in risk-taking behaviors, there is a lack of studies analyzing the relations between both variables. In addition, most of studies usually analyze the effect of sex ratio aggregated to higher levels (e.g., regions or counties). However, operationalizing sex ratio at such high levels of aggregation does not reflect the sex ratio of individuals’ in their closest environment in a precise manner, and therefore the sex of individuals with whom they interact. The object of the present study is to analyze the relation between classroom sex ratio and risk-taking behaviors from a multilevel model, as well as the interaction between life history strategy and classroom sex ratio in male and female adolescent students. Operating sex ratio at classroom level means a closer approximation of sex ratio in individuals’ environments, which can reflect in a more precise manner the real interactions between sexes, as well as the availability of potential partners in their closest environment. Since operational sex ratio theory proposes that skewed sex ratios environments lead to environments of more intense competitions, a relation between risk-taking behaviors in adolescents and classroom sex ratio was expected (cross-level association) (Hypothesis 1). Additionally, given the role of life history strategies on risk-taking behaviors and its calibration to proximate external cues, the relationship between classroom sex ratio and risk-taking behaviors may be different in function of life history strategies. As a result, an interaction between classroom sex ratio and life history strategy on risk-taking behaviors in adolescents was also expected (cross-level interaction) (Hypothesis 2). Figure 1 illustrates the theoretical model.

## Methods

### Participants and Procedure

The sample was composed of 1214 adolescent students nested in 57 classrooms who participated in the study (604 females; 610 males). The average age was 16.15 years (*SD* = 1.23), with range values between 14 and 21 years old, and the nationality of participants was mostly Spanish (91.5%). The study was carried out in six different public secondary education centers, colleges, and vocational training centers in the metropolitan area of Málaga (Spain). The main economic activity in this region is the services sector which is tightly linked to tourism. The socio-economic status of participants was recorded through an open question about the profession of the household member with the highest salary. There were 71.09% valid answers obtained, which were codified and categorized numerically based on the 2011 Classification of Occupations (Spanish National Institute of Statistics, n.d.). Values ranged between 1 (highest SES) and 9 (lowest SES), with a mean of 4.67 (*SD* = 2.50), meaning participants belonged to medium socio-economic levels.

The study was part of a school tutoring activity in which students had to participate and the questionnaires were handed by members of the research team, school counsellors or tutors in the classroom during school hours. Students were explained how to answer questionnaires’ questions and during data collection, no incidences or massive absences of students took place. At a later stage, each education center was given a report with risk-taking behavior indicators for each center. Students’ parents and legal guardians were informed about the objective and method of the study, and they were requested previous informed consent. Ethical approval was granted by the Ethical Committee on Experimentation from the University of Malaga (CEUMA) (Registry number: 45-2018-H).

### Measures

#### Life history strategy

The abbreviated questionnaire Arizona Life History Battery, known as the Mini-K, was used to measure life history strategy (Figueredo et al., 2006). This questionnaire is composed of 20 items that form a single factor and which is interpreted as life history strategy. Higher scores in the Mini-K imply slower life history strategy on a slow/fast continuum. Results were obtained using a Likert-type scale of 5 answers (from *strongly disagree* to *strongly agree*). Each participant’s score was obtained by adding the scores from each item and dividing it by the total number of items. Given the age of participants, the question about the relationship participants had with their children was excluded. The final version with the remaining 19 items showed good internal consistency (α = 0.83).

#### Classroom sex ratio

According to the definition of operational sex ratio, classroom sex ratio was operationalized as the relative number of males compared to females in a specific classroom who participated in the study. Following the recommendations (Ancona et al., 2017), classroom sex ratio was calculated as a proportion (*N*_males_/*N*_males_ + *N*_females_). For each classroom, classroom sex ratio was calculated through the questionnaires, which recorded both the sex and the classroom of each participant. In total, classroom sex ratio was calculated for 57 classrooms. When classroom sex ratio value is greater than 0.5, it means it is male-skewed (i.e., higher proportion of males compared to females in a specific classroom), whereas a score lower than 0.5 means it is a female-skewed classroom (i.e., higher proportion of females compared to males in a specific classroom). If the score is exactly 0.5, then sex distribution is even (same proportion of males and females in a specific classroom). Classroom sex ratio values ranged from 0.10 to 1.00.

Although all students present in classroom participated in the study, researchers could not have access to the actual number of males and females in each classroom, in order to control the respondent rate in each class. However, the Spanish education system mandates that all registered students attend school mandatorily, so school absenteeism is very low and student absences are usually due to exceptional situations (e.g., illness), meaning that the differences between classroom sex ratio values calculated and the actual classroom sex ratios might be considerably minimal.

#### Risk-taking behaviors

The Risky Behavior Questionnaire (RBQ) was used to measure participants’ engagement in risk-taking behaviors. The RBQ is composed of 20 items that encompasses a variety of risky behaviors grouped in one single dimension (Auerbach & Gardiner, 2012): unsafe sexual practices, aggressive and/or violent behaviors, rule breaking, dangerous, destructive, and illegal behaviors, self-injurious behaviors, and substance use. Participants answered on the extent to which they had participated in each behavior in the previous six months with a Likert-type format of four options: (0) Never (not once); (1) Almost never (once a month); (2) Sometimes (2-3 times per month); and (3) Usually (3 or more times per week). Each participant’s score was obtained by adding the scores of each item and dividing it by the total number of items. RBQ’s internal consistency was α = 0.69.

### Statistical Analysis

A multilevel analysis was carried out, where students (level 1) were clustered in classrooms (level 2). Given the recommendations to estimate the effect of life history strategy separated by sex (Copping & Richardson, 2020), analyses were separated by males and females. Thus, the following sequence was carried out for the multilevel models: Model 1 (Model 5, for males), where no predictors were introduced and the objective was to know intra-classroom and between-classroom variability in risk-taking behaviors that would justify the multilevel analysis; Model 2 (Model 6, for males) includes predictors at individual level with the aim of analyzing the relation between life history strategy and risk-taking behaviors; Model 3 (Model 7, for males) includes a predictor at group level to analyze cross-level association between classroom sex ratio and risk-taking behaviors; Model 4 (Model 8, for males) includes cross-level interaction between classroom sex ratio and life history strategy. In males, missing data was only found for life history strategy (*n* = 5), whereas in females missing data was found in life history strategy (*n* = 2) and risk-taking behaviors (*n* = 2). In all cases, the reason for missing data was that these participants did not answered some of the items of the Mini-K or the RBQ. Giving the lower quantity of missing data, cases with missing data were excluded from the data processing. The procedure followed to determine the study’s sample size, all data exclusions (if any), and all measures in the study are reported.

## Results

Table 1 shows descriptive statistics and interrelations of the variables studied based on sex. Only one significant correlation between life history strategy and risk-taking behaviors was found in females. More specifically, females with slower life history strategies related negatively to participation in risk-taking behaviors. However, classroom sex ratio did not show any correlations with life history strategy nor with risk-taking behaviors in female adolescents. In the case of male adolescents, both life history strategy and risk-taking behaviors related negatively. Males with slower life history strategies showed lower participation in risk-taking behaviors, as it was seen in females. Additionally, classroom sex ratio correlated positively to risk-taking behaviors. Thus, male adolescents who belonged to male-skewed classrooms showed higher tendency towards engagement in risk-taking behaviors.

**Table 1:** Means, standard deviations and correlations by sex

	1	2	3	M_females_	SD_females_	M_males_	SD_males_
1. Risk-taking behaviors	−	−0.24***	−0.03	0.48	0.35	0.53	0.39
2. Life history strategy	−0.15***	−	−0.05	0.96	0.41	0.85	0.43
3. Classroom sex ratio	0.15***	−0.04	−	0.45	0.14	0.56	0.16

Above the diagonal: correlations for females; underneath the diagonal: correlations for males

Females: *n* = 604; males: *n* = 610

***p < 0.001, two-tailed

**Fig. 2:**
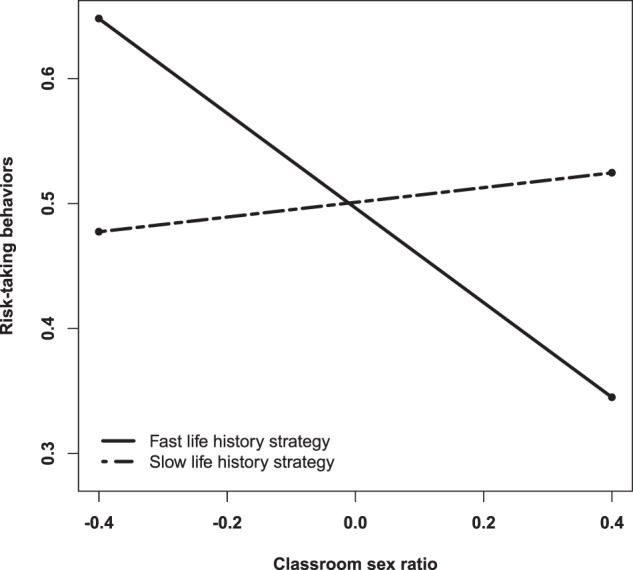
Classroom sex ratio and LHS interaction in females

Table 2 shows results from multilevel models for risk-taking behaviors in female and male adolescents. In females (Model 1-4), Model 1 shows that intra-classroom correlation (ICC) value was 0.09, which indicates that 9% of the variance for risk-taking behaviors in females was caused by differences between classrooms. Likewise, between-classroom variance was 0.011 (*p* ≤ 0.05), while intra-classroom variance was 0.110 (*p* ≤ 0.000). These results showed evidence of a nested data structure, which justifies multilevel modelling. Model 2 included the individual variable of life history strategy (level 1). Life history strategy showed a negative relation with risk-taking behaviors (*γ* = −0.22; *t* = −6.44, *p* ≤ 0.000), meaning that slower life history strategy related to lower engagement in risk-taking behaviors in females. Model 3 included the contextual variable of classroom sex ratio (level 2) and contrarily to what was suggested in Hypothesis 1, classroom sex ratio did not show a relation with risk-taking behaviors in females (γ = −0.02 *t* = −0.14 *p* = 0.886) (Table 2). Finally, Model 4 analyzed the interaction between classroom sex ratio and life history strategy. In line with Hypothesis 2, classroom sex ratio and life history strategy showed interaction over risk-taking behaviors (γ = 0.51; *t* = 2.22, *p* ≤ 0.05). More specifically, a negative relation between classroom sex ratio and risk-taking behaviors was found in females with faster life history strategies. In other words, female-skewed classroom sex ratios related to higher engagement in risk-taking behaviors in females with faster life history strategies. On the other hand, classroom sex ratio and risk-taking behaviors showed a positive relationship in females with slower life history strategies, meaning that male-skewed classroom sex ratio associated, although slightly, with higher engagement in risk-taking behaviors in females with slower life history strategies. Figure 2 shows results from the cross-level interaction between classroom sex ratio and life history strategy on risk-taking behaviors in females.

**Table 2:** Multilevel models tests on risk-taking behaviors in females and males

	Females				Males			
	Model 1 Est. (SE)	Model 2 Est. (SE)	Model 3 Est. (SE)	Model 4 Est. (SE)	Model 5 Est. (SE)	Model 6 Est. (SE)	Model 7 Est. (SE)	Model 8 Est. (SE)
Individual level variables (L1)								
Intercept	0.49 (0.02)***	0.70 (0.04)***	0.70 (0.07)***	0.92 (0.12)***	0.54(0.03)***	0.65 (0.04)***	0.46 (0.09)***	0.51 (0.13)***
LHS		−0.22 (0.03)***	−0.22 (0.03)***	−0.44 (0.11)***		−0.13 (0.04)***	−0.13 (0.03)**	−0.19 (0.12)
Group level variables (L2)								
Classroom sex ratio			−0.02 (0.13)	−0.49 (0.25)^†^			0.34 (0.15)*	0.26 (0.23)
Cross-level interaction								
LHS x classroom sex ratio				0.51 (0.23)*				0.10 (0.21)
Variance components								
Intra-group	0.110	0.102	0.102	0.102	0.126	0.123	0.123	0.123
Between-group	0.011	0.012	0.012	0.012	0.029	0.027	0.024	0.024
Additional information								
ICC	0.09				0.19			
-2*loglikelihood	424.039	382.539	382.519	377.663	538.105	518.359	513.309	513.090
No. estimated parameters	3	4	5	6	3	4	5	6

^†^*p* ≤ 0.1; *p ≤ 0.05; **p ≤ 0.01; ***p ≤ 0.001

In the case of male adolescents, Table 2 shows results from the multilevel models for risk-taking behaviors (Model 5-8). ICC for Model 5 was 0.19, meaning that 19% of the variance in risk-taking behaviors in males was caused by differences between classrooms. The value of between-classroom variance was 0.029 (*p* ≤ 0.001), while the value of intra-classroom variance was 0.126 (*p* ≤ 0.001). These results show evidence of a nested data structure, which also justifies multilevel modelling for males. Model 6 included life history strategy as individual predictor (level 1). As it happened with females, life history strategy showed a negative relation with risk-taking behaviors (γ = −0.13; *t* = −3.81, *p* ≤ 0.000), meaning that slower life history strategy related to lower engagement in risk-taking behaviors in males. Model 7 includes classroom sex ratio (level 2) (Table 2). In line with Hypothesis 1, classroom sex ratio showed an association with risk-taking behaviors in male adolescents. More specifically, male-skewed classroom sex ratio related positively to risk-taking behaviors (γ = 0.34; *t* = 2.30, *p* ≤ 0.05). Finally, the interaction parameter between life history strategy and classroom sex ratio was introduced in Model 8 with the purpose of testing Hypothesis 2. However, interaction between life history strategy and classroom sex ratio was not significant (γ = 0.10; *t* = 0.47, *p* = 0.639).

## Discussion

Risk-taking behaviors are currently among the main public health concerns in adolescents. An evolutionary perspective of risky adolescent behaviors suggests that these behaviors are related to individual life history strategies and sex ratio (Ellis et al., 2012). However, most studies which analyze the relations between sex ratio and risk-taking behaviors operationalize sex ratio at higher levels of aggregation, which does not reflect the actual sex ratio of individuals in their closest environments. Examining the interaction between life history strategies and sex ratio operationalized at a more proximate level is necessary to better understand the influences of these factors in adolescent development, and more precisely their relationship with risk-taking behaviors. In general, findings from the present study show a differential relationship between classroom sex ratio and risk-taking behaviors in male and female adolescents. More specifically, classroom sex ratio related positively to risk-taking behaviors in males, while the relationship between classroom sex ratio and risk-taking behaviors was negative in females with faster life history strategy.

Results regarding the relationship between classroom sex ratio and risk-taking behaviors were not statistically significant in female adolescents. Despite the operational sex ratio theory premise that skewed sex ratio promotes higher competition in the majority sex (Emlen & Oring, 1977), in female adolescents, classroom sex ratio did not affect engagement in risk-taking behaviors. One possible explanation could be females’ tendency to avoid taking part in behaviors that could have a negative impact on offspring survival, which plays a critical role in their ultimate reproductive success (Campbell, 2013). Therefore, the absence of relationship between classroom sex ratio and risk-taking behaviors in females could be due to the potential costs associated with risk-taking behaviors investment being higher than the potential benefits in mating rate (Kokko et al., 2012). By contrast, classroom sex ratio related positively to risk-taking behaviors in male adolescents. More specifically, male-skewed sex ratio (i.e., when males are the majority sex) associated with higher engagement in risk-taking behaviors in male adolescents. On the one hand, this finding, along the absence of relationship between classroom sex ratio and risk-taking behaviors in females, demonstrates that males are more responsive than females to external cues of mating and competition (Ellis, et al., 2012). These results are also in line with the fact that reproductive success in males is more related to accessing potential mates, compared to females, and which promotes greater intrasexual competition (Trivers, 1972). Male-skewed classroom sex ratio generates an environment of male-male competition and courtship behaviors (Weir et al., 2011). From sexual selection theory, in the present study risk-taking behaviors in males could be expressed as a means of showing good genes (Zahavi, 1975) or good provider qualities (Marlowe, 2003).

Findings from the present study also showed that classroom sex ratio interacted with life history strategy in female adolescents. The relation between classroom sex ratio and risk-taking behaviors was higher in females with faster life history strategy than in females with slower life history strategies. Females with faster life history strategies showed higher engagement in risk-taking behaviors in female-skewed classroom sex ratio (i.e., when females are the majority sex), showing the opposite effect in male-skewed classroom sex ratio (i.e., when males are the majority sex). These results suggest that, in a context of a shortage of potential sexual partners, females with faster life history strategies may engage in risk-taking behaviors as a form of social competition and attraction (Clutton-Brock & Huchard, 2013). By contrast, classroom sex ratio showed a lower association with risk-taking behaviors in slower life history strategy females. This relation was negative, meaning that females with slower life history strategies reduced their engagement in risk-taking behaviors in female-skewed classroom sex ratios (i.e., when females are the majority sex). In fact, the scarcity of males can lead some females to have more interest in developing a career than in finding a partner (Durante et al., 2012). As a result, it is possible that in the context of shortage of males, female adolescents with slower life history strategies adjust their investment towards growth instead of reproduction, which in turn would probably imply lower engagement in risk-taking behaviors. This behavioral adjustment in females with slower life history strategies is in line with the greater future expectations of slow life history strategies (Sear, 2020).

Interestingly, classroom sex ratio and life history strategy did not interact in male adolescents over risk-taking behaviors. Although males showed higher variation in risk-taking behaviors than females in relation to life history strategies (Salas-Rodríguez et al., 2021), findings from the present study suggest that sex ratio is not related to this variability in males. These results along with those found in females would be in line with previous studies that suggest that life history strategies are more sensitive to external cues in females than in males (Richardson et al., 2017). Furthermore, this also demonstrates that life history strategies may be affected by proximate contextual cues, in consonance with the concept of phenotypic plasticity (Sear, 2020), although only in females. Findings also show that life history strategy may be calibrated according to external cues in adolescence, beyond unpredictability and harshness, which adds to previous findings in childhood (e.g., Chang et al., 2019) and adulthood (e.g., Hill et al., 2013).

Despite these findings, there are certain limitations to the present study that should be noted. One of those limitations is the fact that answers provided by participants might be biased due to the presence of their peers while completing the questionnaires, which were handed and completed in the classroom. Moreover, given that classroom sex ratio was calculated based on the questionnaires’ answers, the possible absence of some students on the dates the questionnaires were handed is another limitation. This means that classroom sex ratio values obtained might not represent real values in a precise manner. Finally, risky behaviors stated by students can also be carried out outside the school center and, therefore, they would be related to the sex ratio of students’ groups of friends outside school environment.

Despite such limitations, the present study has the strength of operating with a sex ratio at an aggregation level that is closer to the individual compared to previous studies where sex ratio was analyzed at local levels (e.g., nationwide). The ecological fallacy was avoided by obtaining a sex ratio that is more adjusted to the closest environment of individuals. Likewise, by applying multilevel modelling the role of classroom sex ratio was placed at a context level. This multilevel procedure is in line with the biopsychosocial approach of the evolutionary framework of adolescent risky behaviors (Ellis et al., 2012), which considers that adolescents adjust their behaviors based on individual and environmental variables. Finally, the present study also offers an advantage related to the use of life history strategy variable at individual level. More specifically, the interaction found between classroom sex ratio and life history strategy in female adolescents demonstrates the need to analyze the influence of individual variables in the adjustment to proximate sex ratio, as it has been suggested (Schacht et al., 2017). This enabled to reflect the complexity of the relation of sex ratio with risk-taking behaviors in females, and which would help to explain, in part, the contradictory findings observed between sex ratio and risk-taking behaviors. In this sense, it would be interesting to analyze additional individual variables linked to risk-taking behaviors, which could help shed light on the contradictory results obtained on the relation between sex ratio and risk-taking behaviors. For example, it has been observed that seeking status and mating are variables strongly linked to engagement in risk-taking behaviors in male adolescents (Ellis et al., 2012). Future research could focus on analyzing the interaction between sex ratio and both variables as well as their association with risk-taking behaviors.

At practice level, programs aimed at preventing risky behaviors in adolescents should pay special attention to the potential adaptive value of such behaviors. It has been seen that risk-taking behaviors lead to potential benefits for adolescents (e.g., Tomova et al., 2021). Based on the results obtained in the present study, such interventions could also consider how adolescents adjust their engagement in risky behaviors based on classroom sex ratio, as well as their life history strategies, since this is different in males and females. More specifically, when working with male adolescents, intervention programs should consider how in classrooms with lower numbers of females, inter-male competition is more intense, thus leading males to engage more in risky behaviors. In the case of female adolescents, the focus should be placed on females with faster life history strategies in female-biased classrooms. Higher participation in risky behaviors by females with faster life history strategies could be acting as a means of attracting potential partners in a context with low numbers of males. It would be therefore interesting to provide both male and female adolescents in such contexts with healthier and less dangerous alternatives to competitive and mate-attracting behaviors. However, there is a chance that many of these risky behaviors might be inevitable in a way, since adolescents might perceive potential benefits against the costs of such behaviors (e.g., substance use as a way to be accepted by the peer group). Therefore, intervention programs should aim at reducing the potential risks derived from such behaviors to the extent possible (e.g., providing more information and knowledge on substance use).

## Conclusion

Previous studies have demonstrated that sex ratio and life history strategy are important factors related to risk-taking behaviors in adolescents, although the interaction between both variables has not yet been analyzed in depth. Most empirical research operationalize sex ratio aggregated at higher levels (e.g., states), which may not reflect the actual sex ratios of individuals at a more proximate level. This study examined the relation between classroom sex ratio and risk-taking behaviors, along the interaction between classroom sex ratio and life history strategy in male and female adolescents. In females, classroom sex ratio related negatively to risk-taking behaviors in faster life history strategists. More specifically, female adolescents with faster life history strategies showed higher engagement in risk-taking behaviors in female-skewed classroom sex ratios (i.e., when females are the majority sex). In males, classroom sex ratio related positively to risk-taking behaviors, with males showing higher engagement in risk-taking behaviors in male-skewed classroom sex ratio (i.e., when males are the majority sex). Additionally, classroom sex ratio and life history strategy did not interact in males. Thus, sex ratio and life history strategy, and their interaction are fundamental in understanding adolescent development. In essence, these results highlight the potential adaptive value of risk-taking behaviors, which should be considered for the design of intervention programs.
